# Deep learning to predict progression independent of relapse activity at a first demyelinating event

**DOI:** 10.1093/braincomms/fcaf243

**Published:** 2025-07-04

**Authors:** Llucia Coll, Deborah Pareto, Francisco Aparicio-Serrano, Susana Otero-Romero, Alvaro Cobo-Calvo, Evy Reinders, Manel Alberich, María Jesús Arévalo, Georgina Arrambide, Cristina Auger, Joaquín Castilló, Manuel Comabella, Ingrid Galán, Luciana Midaglia, Carlos Nos, Frederik Novak, Arnau Oliver, Jordi Río, Breogán Rodríguez-Acevedo, Jaume Sastre-Garriga, Ángela Vidal-Jordana, Ana Zabalza, Xavier Montalban, Àlex Rovira, Mar Tintoré, Xavier Lladó, Carmen Tur

**Affiliations:** Multiple Sclerosis Centre of Catalonia (Cemcat), and Department of Neurology, Hospital Universitari Vall d’Hebron, Universitat Autònoma de Barcelona, Barcelona 08035, Spain; Section of Neuroradiology, Department of Radiology (IDI), Hospital Universitari Vall d’Hebron, Universitat Autònoma de Barcelona, Barcelona 08035, Spain; Multiple Sclerosis Centre of Catalonia (Cemcat), and Department of Neurology, Hospital Universitari Vall d’Hebron, Universitat Autònoma de Barcelona, Barcelona 08035, Spain; Multiple Sclerosis Centre of Catalonia (Cemcat), and Department of Neurology, Hospital Universitari Vall d’Hebron, Universitat Autònoma de Barcelona, Barcelona 08035, Spain; Multiple Sclerosis Centre of Catalonia (Cemcat), and Department of Neurology, Hospital Universitari Vall d’Hebron, Universitat Autònoma de Barcelona, Barcelona 08035, Spain; Multiple Sclerosis Centre of Catalonia (Cemcat), and Department of Neurology, Hospital Universitari Vall d’Hebron, Universitat Autònoma de Barcelona, Barcelona 08035, Spain; Section of Neuroradiology, Department of Radiology (IDI), Hospital Universitari Vall d’Hebron, Universitat Autònoma de Barcelona, Barcelona 08035, Spain; Multiple Sclerosis Centre of Catalonia (Cemcat), and Department of Neurology, Hospital Universitari Vall d’Hebron, Universitat Autònoma de Barcelona, Barcelona 08035, Spain; Multiple Sclerosis Centre of Catalonia (Cemcat), and Department of Neurology, Hospital Universitari Vall d’Hebron, Universitat Autònoma de Barcelona, Barcelona 08035, Spain; Section of Neuroradiology, Department of Radiology (IDI), Hospital Universitari Vall d’Hebron, Universitat Autònoma de Barcelona, Barcelona 08035, Spain; Multiple Sclerosis Centre of Catalonia (Cemcat), and Department of Neurology, Hospital Universitari Vall d’Hebron, Universitat Autònoma de Barcelona, Barcelona 08035, Spain; Multiple Sclerosis Centre of Catalonia (Cemcat), and Department of Neurology, Hospital Universitari Vall d’Hebron, Universitat Autònoma de Barcelona, Barcelona 08035, Spain; Multiple Sclerosis Centre of Catalonia (Cemcat), and Department of Neurology, Hospital Universitari Vall d’Hebron, Universitat Autònoma de Barcelona, Barcelona 08035, Spain; Multiple Sclerosis Centre of Catalonia (Cemcat), and Department of Neurology, Hospital Universitari Vall d’Hebron, Universitat Autònoma de Barcelona, Barcelona 08035, Spain; Multiple Sclerosis Centre of Catalonia (Cemcat), and Department of Neurology, Hospital Universitari Vall d’Hebron, Universitat Autònoma de Barcelona, Barcelona 08035, Spain; Multiple Sclerosis Centre of Catalonia (Cemcat), and Department of Neurology, Hospital Universitari Vall d’Hebron, Universitat Autònoma de Barcelona, Barcelona 08035, Spain; Research Institute of Computer Vision and Robotics, University of Girona, Girona 17003, Spain; Multiple Sclerosis Centre of Catalonia (Cemcat), and Department of Neurology, Hospital Universitari Vall d’Hebron, Universitat Autònoma de Barcelona, Barcelona 08035, Spain; Multiple Sclerosis Centre of Catalonia (Cemcat), and Department of Neurology, Hospital Universitari Vall d’Hebron, Universitat Autònoma de Barcelona, Barcelona 08035, Spain; Multiple Sclerosis Centre of Catalonia (Cemcat), and Department of Neurology, Hospital Universitari Vall d’Hebron, Universitat Autònoma de Barcelona, Barcelona 08035, Spain; Multiple Sclerosis Centre of Catalonia (Cemcat), and Department of Neurology, Hospital Universitari Vall d’Hebron, Universitat Autònoma de Barcelona, Barcelona 08035, Spain; Multiple Sclerosis Centre of Catalonia (Cemcat), and Department of Neurology, Hospital Universitari Vall d’Hebron, Universitat Autònoma de Barcelona, Barcelona 08035, Spain; Multiple Sclerosis Centre of Catalonia (Cemcat), and Department of Neurology, Hospital Universitari Vall d’Hebron, Universitat Autònoma de Barcelona, Barcelona 08035, Spain; Faculty of Medicine, Universitat de Vic-Universitat Central de Catalunya (UVic-UCC), Vic 08500, Spain; Section of Neuroradiology, Department of Radiology (IDI), Hospital Universitari Vall d’Hebron, Universitat Autònoma de Barcelona, Barcelona 08035, Spain; Multiple Sclerosis Centre of Catalonia (Cemcat), and Department of Neurology, Hospital Universitari Vall d’Hebron, Universitat Autònoma de Barcelona, Barcelona 08035, Spain; Faculty of Medicine, Universitat de Vic-Universitat Central de Catalunya (UVic-UCC), Vic 08500, Spain; Research Institute of Computer Vision and Robotics, University of Girona, Girona 17003, Spain; Multiple Sclerosis Centre of Catalonia (Cemcat), and Department of Neurology, Hospital Universitari Vall d’Hebron, Universitat Autònoma de Barcelona, Barcelona 08035, Spain

**Keywords:** multiple sclerosis, progression independent of relapse activity, predictive model, brain MRI, deep learning

## Abstract

Progression independent of relapse activity is the main cause of irreversible disability in multiple sclerosis and is strongly associated with older age at symptom onset. Early and accurate prediction, at symptom onset, of which patients are at highest risk of progression independent of relapses, is an unmet need. This study aimed to develop a deep learning survival model using only routine MRI acquired at the first demyelinating attack to predict the risk of progression independent of relapses, and assess its ability to improve classical age-adjusted predictions. We analysed a prospective cohort of patients under 50, clinically assessed within three months of symptom onset, with available MRI (T1- and T2-Fluid-Attenuated Inversion Recovery sequences). An independent early multiple sclerosis cohort (≤1 year from symptom onset) from the Multiple Sclerosis Partners Advancing Technology and Health Solutions database (*N* = 32) was used for external validation. Patients were assessed for progression independent of relapse activity, defined as a 6-month confirmed increase in the Expanded Disability Status Scale without relapses. Our deep learning model used EfficientNet to estimate the cumulative probability of progression independent of relapses at 1-year intervals. We employed 5-fold cross-validation for model training and testing, assessing performance with the time-dependent concordance index. We also investigated the optimal cumulative probability threshold for binary risk stratification. The model’s ability to improve a classical Cox regression model was evaluated. Additionally, we identified brain regions most relevant to deep learning-based progression independent of relapse activity predictions using an interpretability algorithm. A total of 259 patients were evaluated, 58 (22%) of whom experienced at least one event of progression independent of relapse activity over a median follow-up of 4.2 years. The deep learning model demonstrated high performance (time-dependent concordance index = 0.72) with an accuracy of 78% in the original cohort and 72% in the external cohort for predicting the risk of progression independent of relapse activity. Incorporating the deep learning-derived cumulative probability of progression independent of relapses significantly improved an age-adjusted Cox regression model, raising Harrell’s C index from 0.62 to 0.74. Interpretability revealed the frontoparietal cortex as a key region in predicting progression independent of relapse activity. In conclusion, our deep learning survival model, based on routine MRI at the first demyelinating attack, can accurately identify patients at high risk of progression independent of relapses and may serve as a valuable tool in clinical practice.

## Teaser

Coll *et al*. demonstrate that a deep learning survival model based solely on conventional brain MRI data acquired at the time of the first demyelinating event can predict the development of progression independent of relapse activity with high accuracy. This model outperforms previously proposed survival models and may have important implications for clinical practice

## Graphical Abstract

**Graphical Abstract:**
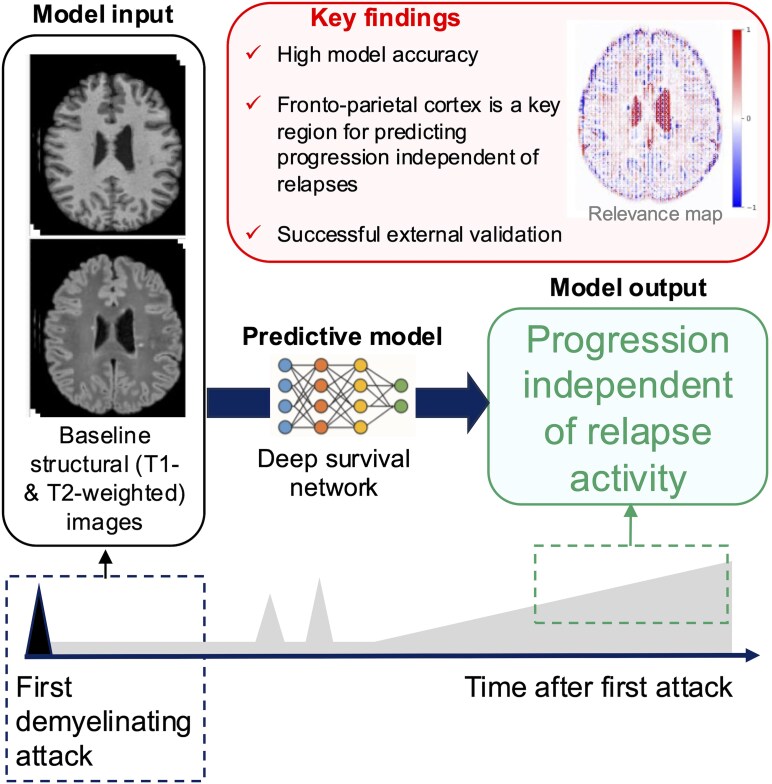

## Introduction

Multiple sclerosis (MS) is a highly disabling inflammatory-demyelinating condition of the central nervous system. In this condition, patients acquire disability through two main mechanisms, progression independent of relapse activity (PIRA), i.e. disability worsening that is not associated with clinical relapses, and relapse-associated worsening.^1^ However, it has been consistently shown that PIRA is the main responsible for the accumulation of disability in this disease in most patients.^2-4^ Furthermore, presenting with a PIRA event after the first clinical attack, especially if such PIRA event occurs early in the disease course, is associated with an unfavourable mid- and long-term prognosis.^4^ This suggests that the development of PIRA reflects the presence of underlying neurodegenerative and chronic inflammatory processes, which probably determine the disease course. Therefore, predicting as early as possible which patients will develop an early PIRA event might have therapeutic implications, especially once new treatments selectively tackling neurodegeneration are approved. Nonetheless, among the factors present at the time of the first attack, only an older age has been unequivocally associated with a greater risk of developing PIRA^3,4^ and, in any case, the predictive ability of older age is only modest.^3,4^ Thus, powerful predictive models of PIRA are still sorely needed.

In the last decades, the increase in computational capacity and the novel algorithms based on deep learning (DL) techniques have allowed us to exploit more deeply and comprehensively the information that MRI offers, beyond pre-extracted MRI measurements such as the number of brain and spinal cord T2 lesions.^5^ So, DL models applied to MRI have been key in improving the diagnosis and prognosis of several neurological conditions, including MS^5^ and other neurodegenerative conditions.^6,7^ Interestingly, DL has also been applied to other types of paraclinical data, such as EEG-based features, with important implications for the diagnosis of neurological and psychiatric conditions.^8-11^

In MS, DL models have been explored to improve the diagnosis of patients and their stratification according to disease stages, while providing the most relevant areas in the brain to make such predictions, shedding important light onto disease mechanisms.^12-14^ Recently, there has been a growing interest in developing image-based DL models to predict disease progression especially at early stages of the disease^15-17^ (please see Supplementary Table 1 for more detailed information). However, long-term predictive DL-based models are still unexplored in MS.

In this study, we aimed to assess whether a DL model was able to predict the risk of PIRA after a first demyelinating attack in a unique, deeply phenotyped cohort of patients and using only information from conventional brain MRI acquired in clinical practice. So, through building DL-based survival models, we investigated which patients were at highest risk of reaching a first PIRA event after a first clinical attack suggestive of MS. Given the relatively limited ability of classical predictive models to forecast PIRA at the first attack, and the potential of DL to uncover subtle yet crucial relationships between MRI features and clinical outcomes, we hypothesized that a DL-based model would provide highly accurate predictions of the risk of developing PIRA, surpassing classical survival models. Indeed, with this study we also aimed to investigate whether our DL-based estimations could improve classical statistical survival models of time to PIRA based on age, the strongest predictor of PIRA so far, and other relevant predictors. Additionally, as a more exploratory aim, we also looked to interpret the reasoning behind the decisions made by our DL model, through explainable artificial intelligence methods. Our underlying hypothesis in that regard was that those areas most relevant for our DL model to make the final decisions would be mainly related to grey matter (GM) pathology, given the strong associations found between brain cortical atrophy and a greater risk of PIRA.^18^ Finally, we aimed to apply our DL-based survival model to a completely unseen cohort of early MS and assess its sensitivity and specificity to identify patients at particularly high risk of reaching PIRA.

## Materials and methods

### Patients

#### Original (training and testing) cohort: Vall d’Hebron University Hospital cohort

This study consists of a retrospective analysis of the prospectively followed up Barcelona deeply phenotyped clinically isolated syndrome/early MS cohort, from the Vall d’Hebron University Hospital (VHUH). This is an ongoing cohort which started in 1995^19^ and includes patients with a first demyelinating attack visited at the Multiple Sclerosis Centre of Catalonia (Cemcat) within 3 months of symptom onset.

Patients with a first demyelinating attack are included in this cohort if they had been clinically evaluated in our centre and had undergone a brain MRI scan within the first 5–6 months after symptom onset. Additionally, patients’ age at first attack had to be 18–50 years and their neurological symptoms and signs could not be attributed to any other neurological disease.^19^

For the current study, we specifically assessed patients included between 2009 and 2022 and established an additional set of inclusion criteria: (i) availability of T1-weighted (T1-w) and transverse T2 fluid-attenuated inversion recovery (T2-FLAIR) sequences at the time of the first attack for image analysis; (ii) at least three clinical assessments (on the expanded disability status scale [EDSS]); (iii) for patients without PIRA, a minimum follow-up of 4 years was required.

#### External validation cohort: MS PATHS cohort

To ensure the generalizability of our proposed model, we carried out an external validation analysis using a cohort of completely unseen patients with early MS (<1 year of disease duration) who belonged to the large Multiple Sclerosis Partners Advancing Technology and Health Solutions (MS PATHS)^20^ database. Additional inclusion criteria for this particular study were: (i) availability of T1-w and T2-FLAIR images for analysis carried out within the first 12 months of symptom onset, with a tolerability period of ±2 months; (ii) at least 2 clinical assessments; (iii) at least 3 years of clinical follow-up after the baseline MRI.

Of note, although the VHUH participated in the MS PATHS initiative, our validation cohort did not include any VHUH patient.

#### Ethical requirements and permissions

Approval was received from the Vall d’Hebron Institute of Research’s Ethics Committee (XMG-INT-2014-01; PR(AG)389/2021) and informed consent was obtained from each patient conforming the cohort.

### Clinical assessments and outcomes

#### Original cohort

All patients were assessed on the EDSS and relapses at each visit, i.e. every 6–12 months or more often, if they developed new symptoms and/or relapses, as per routine practice. We then retrospectively investigated the presence of PIRA, as previously described,^1,2,4^ i.e. as a 6-month confirmed disability worsening (CDW) event which occurred in a relapse-free period. Relapse-free periods were considered as those periods between relapses, starting at least 3 months after any relapse (or 6 months after the first attack) and finalizing at the time of the next relapse. We considered a CDW event as an increase in the EDSS^21^ of 1.5, 1.0, or 0.5 points if the reference (baseline or re-baseline, i.e. after last relapse) EDSS score was of 0, 1.0–5.0, or >5.0, respectively. The reference EDSS score was the first EDSS score obtained at least 3 months after any relapse (or 6 months in the case of the first attack). Such reference EDSS score could never be lower than the very first EDSS score recorded at least 6 months after the first demyelinating attack.^4^

#### External validation cohort

All included patients from the MS PATHS cohort were assessed on the Patient Determined Disease Steps (PDDS),^22^ a patient-reported outcome, which has been proven to have a strong correlation with the EDSS score. Information on self-reported relapses over the previous 12 months was also collected from all patients. This means that the exact relapse dates were not available. For this reason, a formal evaluation of PIRA (as described in the literature) was not possible and we used an alternative (self-reported) PIRA definitions as follows: increase of ≥1 point in the PDDS^23^ between baseline (i.e. MRI acquisition date) and the last clinical follow-up, without any self-reported relapse on the same time-window as this PDDS increase happened.

### MRI data

#### Original cohort

Brain MRI data was acquired as part of clinical practice in our centre with four different magnets from the same vendor (Siemens AG) at two magnetic fields, 1.5T and 3T, using a standardized acquisition protocol.^14^

All T1-w and T2-FLAIR sequences were pre-processed with (i) bias correction,^24^ (ii) skull-stripping,^25^ (iii) registration to MNI152 space, as well as co-registration of T2-FLAIR sequences to T1-w space,^26^ and (iv) min–max voxel intensity normalization. Afterwards, for descriptive purposes, we obtained volumetric MRI measures in native-space T1-w scans. For their calculation, we performed brain structures segmentation^27^ on previously lesion filled^28^ T1-w scans, to obtain the white matter (WM), GM and total intracranial volume fractions. We also performed automatic lesion segmentation^29^ to obtain brain lesion loads.

Additionally, the number of spinal cord lesions and the number of brain lesions were obtained from the radiology report.

#### External validation cohort

All patients from the external cohort were scanned in a 3T Siemens MRI scanner at study baseline (Siemens).^20^ All MRI scans were pre-processed in the same way as the training dataset and used only for inference, with the previously mentioned label definitions.

### Data analysis

#### Descriptive statistics

All variables are described in terms of mean (standard deviation [SD]) or median (range), depending on whether they were normally distributed or not. We compared the main demographic and clinical variables at baseline between patients that reached the event (PIRA) and patients who did not. For that, we used χ^2^ tests and independent-sample *t*-tests, as appropriate, considering as significant a *P*-value < 0.05.

#### DL model to predict time to PIRA

**Figure 1:**
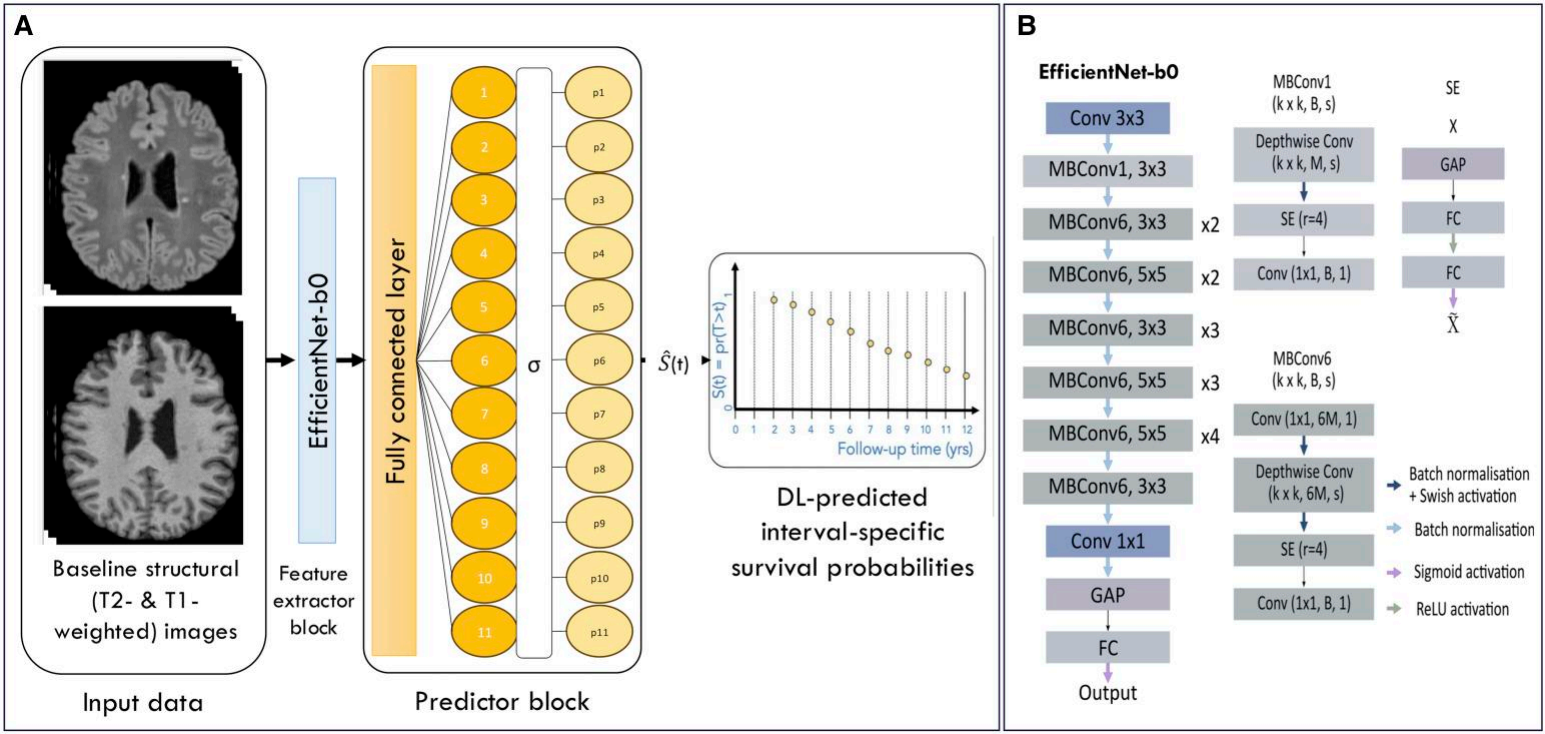
**Study design.** DL model. (**A**) Overview of the proposed pipeline for the prediction of survival probabilities and their evaluation. The T2-FLAIR and T1-w 2D slices are used as input to the pre-trained EfficientNet-b0, whose extracted features are grouped in the discrete predictor to predict the discrete survivor function $\hat{S}(t),\;$ that is, the time-discrete interval-specific survival probabilities, i.e. the probabilities of not reaching PIRA. The predictor block consists of a fully connected layer, which outputs an 11-dimension vector, one for each time interval, followed by a sigmoid activation function, which outputs 11 (interval-specific) survival probabilities. These survival probabilities conform the survivor function, $\hat{S}(t),\;$ representing the probability of not reaching PIRA at each discrete time *t* (at each time interval). Then, the cumulative probability of having had the PIRA event by time *t*, i.e. the *cumulative distribution function*  $\hat{F}(t)$, is obtained as $1-\hat{S}(t).\;$ These interval-specific cumulative probabilities of reaching the event PIRA are then used to find the best threshold to identify, which patients will finally develop the event. Also, these interval-specific probabilities of PIRA are added as covariates in a Cox Proportional Hazards model predicting time to first PIRA (see the Material and methods section for more details). (**B**) EfficientNet-b0 architecture and the main layers forming the convolutional and residual blocks. Conforming the MBConv block there were depth-wise convolutional layers, which expand features instead of reducing them. After that, SE blocks were used to improve the quality of representations by explicitly modelling the inter-dependencies between the channels. Additionally, in between these two blocks a Swish activation was used, defined as *f*(*x*) = *x* · Sigmoid(*x*), that tends to work better than ReLU on deeper models. B, output feature maps; Conv, convolutional layer; DL, deep learning; FC, fully connected; GAP, global adaptive pooling; k, kernel size; M, input feature maps; MBConv, mobile inverted bottleneck; PIRA, progression independent of relapse activity; r, reduction ration of SE; s, stride; SE, squeeze-and-excitation; T1-w, T1-weighted image; T2-FLAIR, T2-weighted fluid-attenuated inversion recovery.

Using the VHUH cohort, we built a DL discrete-time survival model to predict time to PIRA (Fig. 1). The network architecture follows a classical structure of a classification model: feature extractor and predictor block.

As a feature extractor, we adopted the EfficientNet-b0,^30^ a 2D convolutional neural network architecture, pre-trained on large-scale datasets such as ImageNet, which we only had to fine-tune for our problem. Instead, pre-trained 3D convolutional neural networks are less common and usually are pre-trained on smaller medical datasets, which may lead to suboptimal performance. Furthermore, 2D convolutional neural networks have fewer parameters than 3D ones, which were desirable in our case, given the relatively small size of our dataset, to reduce the risk of overfitting. Among the 2D-CNNs, we chose EfficientNet instead of other popular architectures such as ResNet or visual geometry group because of its unique compound scaling method that optimizes the network’s depth, width, and resolution simultaneously, allowing it to achieve better accuracy with fewer parameters than other architectures. The EfficientNet-b0 architecture was composed of a first convolutional layer and successive mobile inverted bottlenecks (MBConv)^31^ with squeeze-and-excitation (SE) optimization,^32^ followed by a last 1 × 1 convolutional layer, a global average pooling (GAP) and a fully connected layer activated with Sigmoid to obtain the different output probabilities (Fig. 1).

So, our predictor block was composed of a fully connected layer that outputs an *n*-dimensional vector (*n* = 11 in our case, corresponding to the equidistant 1-year time intervals, starting from 1 year—i.e. the first time interval was that interval from year 1 to year 2 of follow-up) followed by a Sigmoid activation, giving the probability of surviving (not reaching PIRA) at each evaluated time interval *t*, i.e. the survivor function $\hat{S}(t)$:


(1) $$\hat{S}(t)=pr(T>t)$$

being *T* the actual survival time. Then, from this we obtained the *cumulative probability distribution function*  $\hat{F}(t)$ as $1-\hat{S}(t)\;$, since:


(2) $$\hat{S}(t)=1-\;\hat{F}(t)$$

being $\hat{F}(t)$ the cumulative probability of having had the event (PIRA) by time *t:*


(3) $$\hat{F}(t)=pr(T\leq t)$$

#### Training procedure

A 5-fold cross-validation strategy was used to train and test the proposed model. We sampled the folds to have a similar event-time distribution in each one. In each iteration, 3-folds were used for training (156 scans), 1-fold (52 scans) for validation and the remaining one for testing. From each baseline scan (T1-w and T2-FLAIR scans) we used the central 40 axial slices as input to the 2D end-to-end DL model. This decision was taken after comparing the models’ performance using different samplings, which included the total number of slices, 100, 60 and 40 slices (data not shown). We trained the model using the pre-trained weights on the ImageNet^33^ natural image dataset. At the sixth convolutional block, we ‘unfroze’ the weights and fine-tuned the last convolutional block parameters on our specific dataset, followed by the training of the predictor. The model was trained using the negative log likelihood loss function. This loss is evaluated at each interval, taking into account all input-patient that had the event (or censoring) later than the beginning of that interval.^34^ We trained each fold-model for a maximum of 150 epochs, with a fixed batch size of 64 and an early stopping strategy based on the validation loss behaviour to prevent overfitting, halting training if no improvement was observed for 7 consecutive epochs. The model was optimized with Adam^35^ with an initial learning rate of λ=10–5 and a cosine annealing schedule.^36^

#### Model inference

For inference, we used the same sampling as for training, passing through the model each single 2D slice. After that, the median of the 40 slices (from each patient) was taken as final patient-output. Thus, for every patient we obtained a predicted survival function, from which we obtained the cumulative probability at each time interval to reach a first PIRA event (see Equations 1–3).

#### Evaluation of model performance

We used two different time-dependent accuracy metrics: (i) the extended version of the widely used Harrell's concordance index or c-index^37^: the time-dependent concordance index (c^td^)^38^; (ii) the integrated Brier score (IBS),^39^ which quantifies the mean square difference between the predicted survival probabilities and the observed event-time. That is, the IBS is a quantitative measure of how different the estimated survival function (through our DL model) and the empirical survival function (obtained through Kaplan–Meier curves^40^) are. The IBS is computed at each time interval, and we report the mean (SD) for the whole study period. The smaller the IBS, the better the predictions are. For a model with an incidence of outcome of 50%, the maximum score (non-informative) is 0.25 and 0 a perfect model.^39^ Since our outcome incidence is 22%, we will consider acceptable IBS values between 0 and 0.17, calculated from 0.22 × (1–0.22)^2^ + (1–0.22) × 0.22^2^.^39^

Additionally, we computed the receiver operating characteristic (ROC) curves for the DL-based interval-specific cumulative probabilities of presenting a PIRA event. Areas under the ROC curve (AUC) were formally compared through χ^2^ tests, correcting for multiple comparisons (Šidák correction). Afterwards, using the interval-specific cumulative probability of PIRA with the highest ROC AUC value, we estimated the threshold which offered the highest accuracy to predict the event (i.e. *best VHUH threshold*). Sensitivity and specificity values as well as positive and negative predictive values for such threshold are reported. This *best VHUH threshold* was then tested in the external validation cohort.

#### Assessment of the added value of the DL-based estimations to conventional survival models of time to PIRA

We first built the best possible Cox proportional hazards (Cox PH) regression model for time to first PIRA event based on the literature,^19^ considering, as predictors, age at the first demyelinating attack, sex, brain and spinal cord lesion numbers, and CSF OBs at first attack. These were only kept if *P* < 0.10. Adjusted hazard ratios (aHR) with 95% Confidence Intervals (95% CI) were obtained for each predictor. This best possible model was used as a benchmark and, also, this is the model that we then tried to improve by adding the DL-based probability of PIRA. So, finally, the most accurate DL-based interval-specific cumulative probabilities of a first PIRA event (i.e. that with highest ROC AUC in the previous step) was entered singly in the best predictive model previously obtained. If the newly added DL-based interval-specific probability of PIRA was significant (*P* < 0.05), we assumed it provided independent information and, therefore, improved the classical Cox PH model.

Cox PH models’ accuracies were assessed through the concordance index (Harrell’s C index). For all survival models, the proportional hazards assumption was assessed through visual inspection of scaled Schoenfeld residuals and through scaled Schoenfeld residuals test.

#### Assessing model interpretability through relevance maps: deep SHAP

In our study, the interpretability of our model predictions was crucial. To achieve this, we used the SHapley Additive exPlanations (SHAP) framework.^41^ The essence of SHAP lies in assigning relevance or importance values to individual features, such as pixels in our context, with respect to the model’s output prediction. In a classification scenario, the SHAP framework attributes positive or negative contributions to different features, indicating their impact on the predictive class. However, since we are dealing with a regression task we align the positive values with an increase in the network’s output (i.e. increased survival) and the negative values with a reduction (i.e. decreased survival, so, increased chances of developing PIRA). So, we used the DeepExplainer method within the SHAP framework to quantify the importance of each pixel’s contribution to our model’s prediction of PIRA survival.^41^ The resulting SHAP value maps are presented on a blue-to-red scale. Negative relevance, indicated by the colour blue, suggests a higher likelihood of encountering a PIRA, while positive relevance, shown in red, suggests a decreased probability of encountering a PIRA, thereby indicating a greater chance of survival. Once the individual SHAP maps for each patient were obtained, we computed a population-average map to reveal to which regions the model was paying more attention while performing the prediction. For that purpose, we multiplied the SHAP maps, which were previously normalized and binarized through applying a threshold set at 95% percentile of positive and negative relevance, by a brain parcellation map. This allowed us to identify the most relevant brain anatomical areas associated with the risk of experiencing a first PIRA event.

#### External validation analysis

Included patients were tested using our DL survival model, without any re-training or calibration, obtaining their cumulative probability of reaching a PIRA for each 1-year interval period of the external cohort (the time intervals had a length of 1 year, as in the original cohort). Since in the original cohort, we had trained five DL models in parallel (one for each fold of the study sample), all five models were applied to the external cohort. Thus, all patients had five estimated probabilities of PIRA for each time interval, i.e. one for each model, which were then averaged to obtain one cumulative probability of PIRA for each time interval. Afterwards, the *best VHUH threshold* was applied to the corresponding interval-specific cumulative probability in the external cohort. That is, we applied the *best VHUH threshold* to the probability of PIRA for the same time interval as that selected in the training and testing cohort for having the highest AUC. Accuracy, sensitivity, specificity and positive and negative predictive values for that *best VHUH threshold* were obtained.

#### Implementation details

The proposed DL method and data analysis were entirely implemented in Python, using the Pytorch library.^42^ For the evaluation metrics we used the implementations in pycox^43^ and lifelines python package.^44^ We ran all DL experiments on a GNU/Linux machine box running Ubuntu 20.04, with 128 GB RAM. For training and testing the model, we used a single Quadro RTX 5000 GPU (NVIDIA Corp, USA) with 16GB VRAM memory. Conventional Cox PH models were fitted in Stata/SE 14.2 for Mac (64-bit Intel).

A glossary of key terms used to describe the analyses performed in this paper is provided in Supplementary Table 2.

## Results

### Descriptive statistics (original cohort)

**Figure 2:**
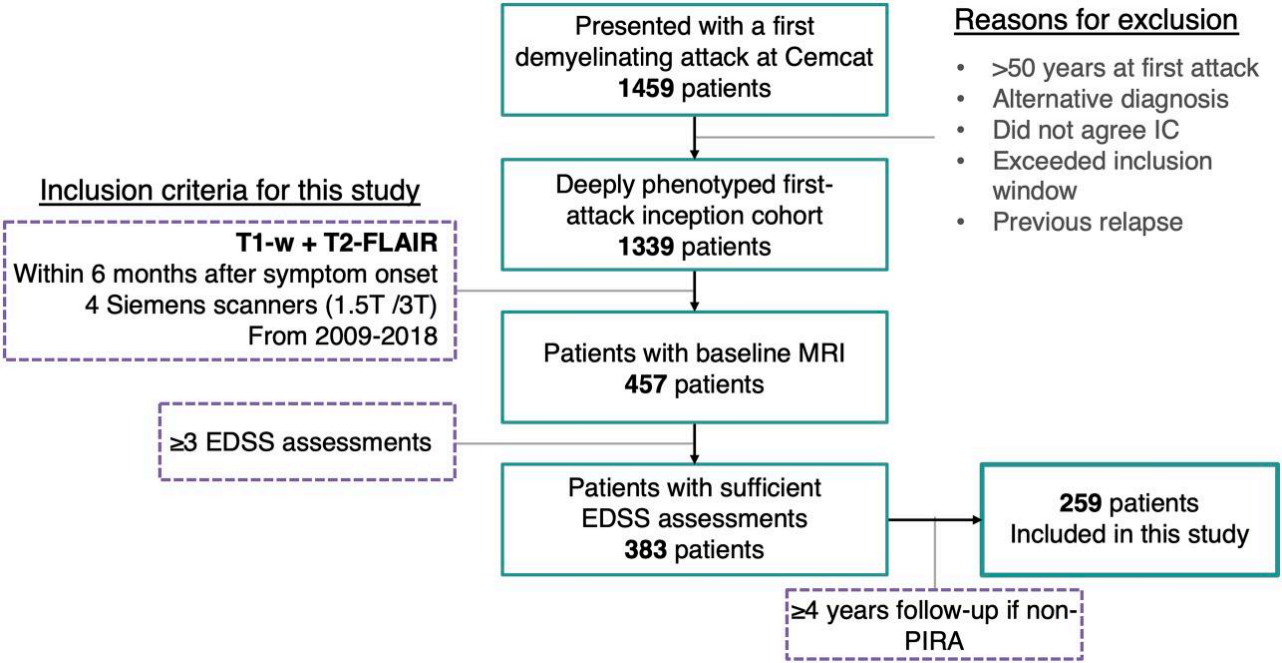
**Patient flowchart.** EDSS, expanded disability status scale; IC, informed consent; PIRA, progression independent of relapse activity.

We included 259 patients (170 female, mean age: 34.3 [range: 19; 50] years) with a first demyelinating attack, who fulfilled the criteria (Fig. 2). Of those, 58 (22%) had at least one PIRA event at a median follow-up time of 4.2 years (range 1.7; 12.3). Median follow-up times for PIRA and non-PIRA patients were, respectively, 7.8 (3.1; 12.6) and 7.7 (4.1; 12.7) years. At the time of the first attack, there was some borderline evidence of patients who later developed PIRA being older (36.2 versus 33.8 years, *P* = 0.06) and having higher lesion volumes (4.8 versus 3.1 mL, *P* = 0.05) than those not developing it. No other significant differences were observed at the time of the first attack. In both cohorts, the most common topography of the first attack was the optic nerve (38% and 39%, for PIRA and non-PIRA groups, respectively: *P* = 0.676) and most patients presented with positive oligoclonal bands in the CSF (71% and 60%, respectively: *P* = 0.178). At the last visit, patients who had developed PIRA at any time during the follow-up showed higher EDSS scores than patients without PIRA (2.0 versus 1.0, *P* < 0.001), but no other differences were observed. Table 1 summarises all the baseline and follow-up data.

**Table 1:** Original cohort: baseline and follow-up characteristics

	Full cohort	PIRA	Non-PIRA	*P*-value
*N*, (%)	259	58 (22)	201 (78)	
Baseline characteristics				
Female, *n* (%)	170 (66)	40 (69)	130 (65)	0.545
Age at first attack, years, mean (SD)	34.3 (7.9)	36.2 (8.2)	33.8 (7.8)	0.048
EDSS at first visit, median [range]	1.5 [0.0, 4.5]	1.5 [0.0, 4.5]	1.5 [0.0, 3.5]	0.999
First-attack topography, *n* (%)				0.676
Brainstem	49 (19)	13 (22.4)	36 (17.9)	
Optic nerve	100 (39)	22 (37.9)	78 (38.8)	
Spinal cord	81 (31)	17 (29.3)	64 (31.8)	
Polyregional	12 (5)	1 (1.7)	11 (5.5)	
Other	17 (6)	5 (8.6)	12 (6)	
Presence of OB, *n* (%)				0.178
Positive	142 (63)	36 (71)	106 (60)	
Negative	85 (37)	15 (29)	70 (40)	
Brain lesions, *n* (%)				0.320
0	60 (23)	10 (17)	50 (25)	
1–3	28 (11)	4 (7)	24 (12)	
4–8	43 (16)	10 (17)	33 (16)	
≥9	128 (49)	34 (59)	94 (47)	
Spinal cord lesions, *n* (%)				0.720
0	126 (49)	27 (46.6)	99 (49.3)	
1	54 (21)	14 (24.1)	40 (19.9)	
2–3	27 (10)	6 (10.3)	21 (10.4)	
>3	34 (13)	9 (15.5)	25 (12.4)	
Unknown	18 (7)	2 (3.4)	16 (8.0)	
Brain lesion load, ml, mean (SD)	3.40 (4.3)	4.80 (6.3)	3.10 (3.4)	0.050
Brain GM fraction, mean (SD)	0.43 (0.02)	0.43 (0.03)	0.43 (0.03)	0.860
Brain WM fraction, mean (SD)	0.37 (0.02)	0.37 (0.02)	0.37 (0.02)	0.900
Brain parenchymal fraction, mean (SD)	0.80 (0.02)	0.80 (0.03)	0.80 (0.02)	0.940
Scanner model, *n* (%)				0.890
Tim Trio	236 (91.1)	53 (91.4)	183 (91)	
Symphony	12 (4.6)	3 (5.2)	9 (4.5)	
Avanto	9 (3.5)	2 (3.4)	7 (3.5)	
Symphony Tim	2 (0.8)		2 (1)	
Follow-up characteristics				
Total FU time, years, median (range)	7.70 [3.1, 12.7]	7.80 [3.1, 12.6]	7.70 [4.1, 12.7]	0.950
Disease duration at first PIRA event, years, median (range)	4.20 (1.7; 12.3)	4.20 (1.7; 12.3)		
EDSS at last FU, median (range)	1.0 (0.0; 6.0)	2.0 (0.0; 6.0)	1.0 (0.0; 5.0)	<0.001
Annualized relapse rate, mean (SD)	0.25 (0.18)	0.23 (0.16)	0.26 (0.19)	0.220
DMT treated during FU, *n* (%)	161 (62)	41 (71)	120 (60)	0.170
Proportion of time DMT treated, mean (SD)	0.53 (0.43)	0.58 (0.39)	0.51 (0.44)	0.240

DMT, disease-modifying treatment; FU, follow-up; GM, grey matter; OB, oligoclonal bands; PIRA, progression independent of relapse activity; SD, standard deviation; GMF, grey matter fraction; WMF, white matter fraction; BPF, brain parenchymal fraction.

### DL prediction of PIRA

**Figure 3:**
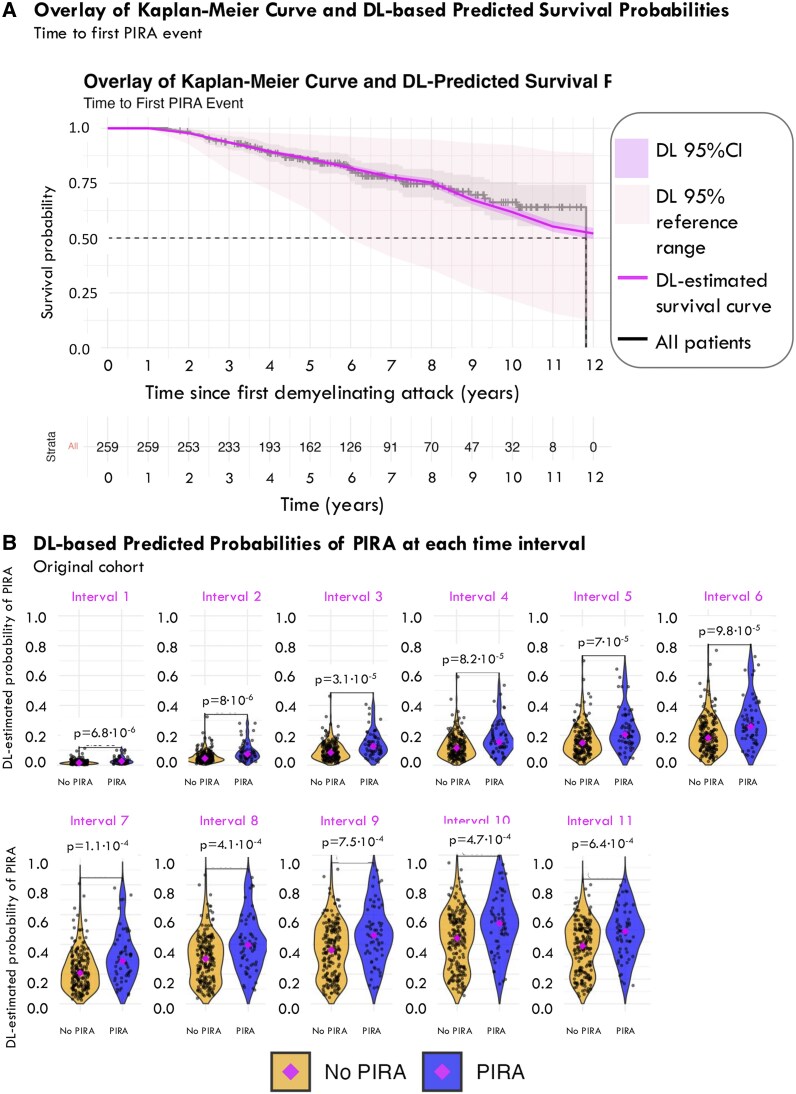
**DL-based predictions in the original cohort.** (**A**) Overlay of Kaplan–Meier curve (grey) and DL-based survival predictions of PIRA (pink line) in the original cohort. The darker pink ribbon represents the 95% CI of the estimation, whereas the lighter pink one represents the 95% reference range, i.e. the DL-predicted PIRA survival probabilities in the population, from the 2.5th to the 97.5th percentiles. As observed, the DL-based predictions accurately mirrored the Kaplan–Meier curve, especially from symptom onset until the 8th year of follow-up. The mean time-dependent concordance index (c^td^) across folds was 0.72 (range 0.68 to 0.78) and the mean IBS was 0.1 (SD 0.04). (**B**). DL-based predicted probabilities of PIRA at each time interval. Here, each dot in the graph represents the predicted probability of developing PIRA. Patients who developed PIRA during the follow-up had, on average, greater DL-predicted risk of developing PIRA at all time points: Mann–Whitney U-test (for comparison of medians): Interval 1: *z* (test statistic, large-sample normal [*Z*] approximation of the U statistic) = −4.501, *P* = 6.8·10^−6^; Interval 2: *z* = −4.465, *P* = 8·10^−6^; Interval 3: *z* = −4.168, *P* = 3.1·10^−5^; Interval 4: *z* = −3.940, *P* = 8.2·10^−5^; Interval 5: *z* = −3.977, *P* = 7·10^−5^; Interval 6: *z* = −3.896, *P* = 9.8·10^−5^; Interval 7: *z* = −3.860, *P* = 0.00011; Interval 8: *z* = −3.534, *P* = 0.00041; Interval 9: *z* = −3.373, *P* = 0.00075; Interval 10: *z* = −3.500, *P* = 0.00047; Interval 11: *z* = −3.400, *P* = 0.00064. Sample size for all comparisons: *N* = 259. DL, deep learning; IBS, integrated Brier score; SD, standard deviation; PIRA, progression independent of relapse activity.

Our DL survival model successfully predicted the risk of reaching a first PIRA event with a mean ctd across folds of 0.72 (range 0.68 to 0.78) and a mean IBS of 0.1 (SD 0.04), reflecting the model’s accuracy. Such an accuracy was particularly high up to 8-years follow-up period, when the DL-based survival estimates started to differ from the empirical survival function (Fig. 3A).

The median interval-specific cumulative probabilities of reaching a first PIRA event for each time interval were always significantly greater for patients who finally developed PIRA than for those never developing it (Fig. 3B). The actual distribution of DL-based interval-specific cumulative probabilities of reaching a first PIRA event are shown in Supplementary Fig. 1.

#### Best threshold to predict PIRA

**Figure 4:**
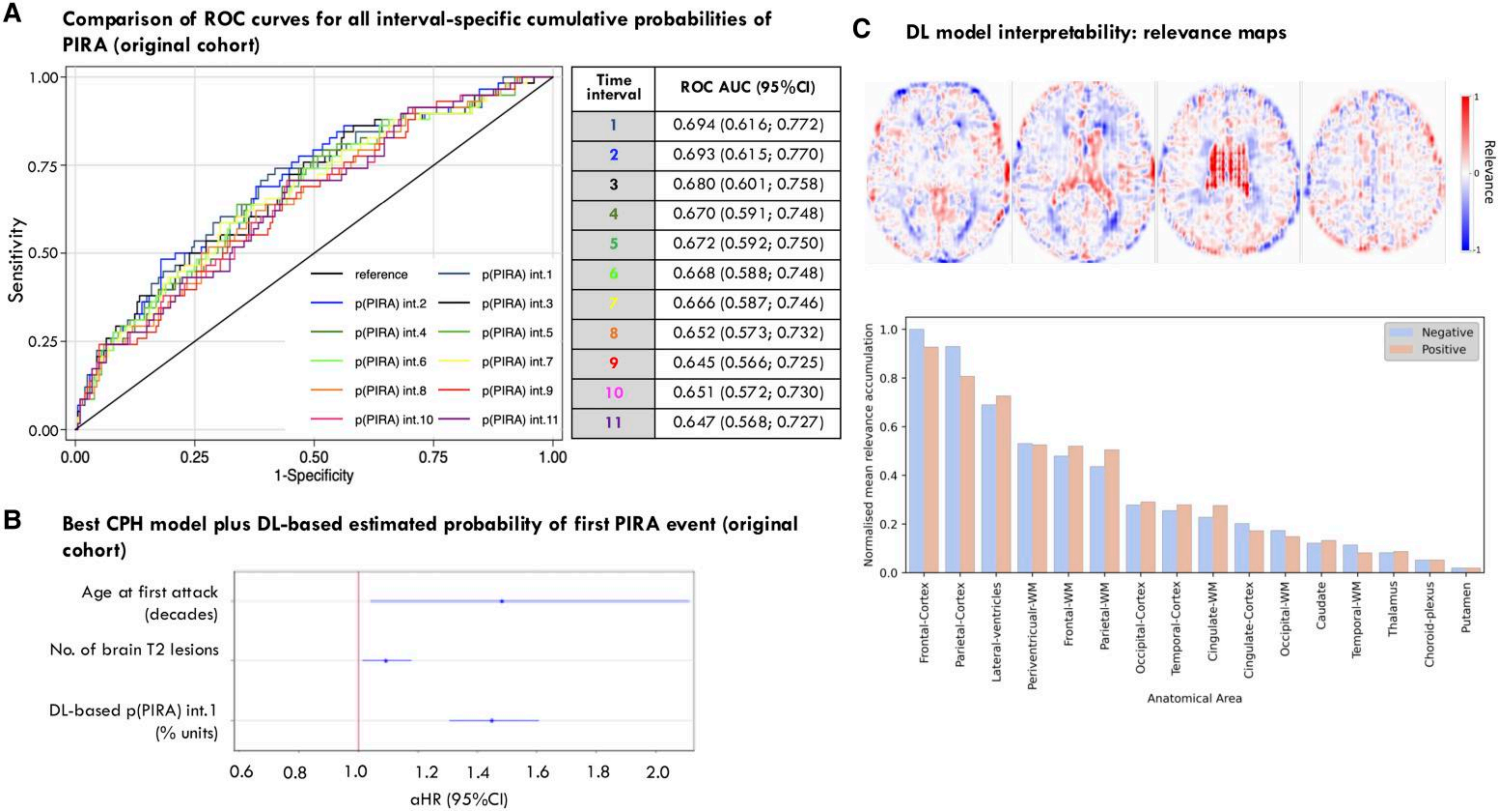
**Assessment of DL model performance and interpretability.** (**A**) Comparison of ROC curves for the different interval-specific cumulative probability of a first PIRA event: the AUC for the first interval-specific probability is the greatest, although the difference with the rest of the curves did not reach statistical significance according to a χ^2^ test, correcting for multiple comparisons (Šidák correction): χ^2^ statistic (10 degrees of freedom) = 17.32, *P* = 0.0676. Sample size: *N* = 259. (**B**) Coefficient plot showing the independent contribution of age and lesion load at first attack plus the DL-based cumulative probabilities of PIRA, estimated at the first time interval, to predict PIRA through a Cox PH model (and Wald tests to assess individual covariate effects): age at first attack (in decades): aHR = 1.482 (1.041; 2.111), *z* (test statistic) = 2.18, *P* = 0.029; number of brain T2 lesions at first attack: aHR = 1.093 (1.014; 1.179), *z* = 2.31, *P* = 0.021; DL-based cumulative probabilities of PIRA: aHR = 1.449 (1.306; 1.607), *z* = 7.01, *P* < 0.001. Sample size for all analyses: *N* = 259. (**C**) Population-average maps of SHAP values, i.e. relevance maps (top row) and the most relevant areas for predicting PIRA (bottom row). Most relevant regions for PIRA prediction were the frontal and parietal cortex, lateral ventricles, periventricular, frontal and parietal WM. Cortical areas had a more negative relevance, lateral ventricles a more positive one, while a similar relevance value was observed in periventricular WM. Sample size for all analyses: *N* = 259. 95% CI, 95% confidence interval; aHR, adjusted hazard ratio; AUC, area under the ROC curve; Cox PH, Cox proportional hazards; DL, deep learning; PIRA, progression independent of relapse activity; ROC, receiver operating characteristic; WM, white matter.

When the different interval-specific cumulative probabilities of reaching a first PIRA event were individually assessed as potential classifiers through ROC curves, we found that the probability at the first interval was the most accurate one: the AUC was greater for the first interval (0.6940 [95% CI 0.61642 to 0.77164]) than for the others (AUC between 0.6454 and 0.6925), although the difference did not reach statistical significance (*P* = 0.0676) (Fig. 4). Based on that ROC curve (of the cumulative probability of PIRA for the first interval), the estimated best (i.e. most accurate) threshold to be later applied to the external cohort was 0.03901. So, when we divided our population based on whether *p*(PIRA) interval 1 ≥ 0.03901 or <0.03901, we obtained an accuracy of 78.38%, a sensitivity of 25.86%, and a specificity of 93.53%. Table 2 shows full details on the evaluation metrics.

**Table 2:** Evaluation metrics of the best threshold for the DL-based probability of PIRA

	Original cohort *N* = 259		External validation cohort *N* = 32	
DL survival model				
Mean c^td^ across folds (range)	0.72 (0.68 to 0.78)		NA^a^	
IBS (SD)	0.1 (0.04)		NA^a^	
**Threshold: DL-based probability of PIRA for first interval ≥ 3.901**				
**Metric^b^**	**Estimate**	**95% CI**	**Estimate**	**95% CI**
Sensitivity	25.86%	15.26% to 39.04%	25.00%	0.63% to 80.59%
Specificity	93.53%	89.19% to 96.51%	78.57%	59.05% to 91.70%
Positive predictive value	53.57%	33.87% to 72.49%	14.29%	0.36% to 57.87%
Negative predictive value	81.39%	75.76% to 86.19%	88.00%	68.78% to 97.45%
Accuracy	78.38%	72.86% to 83.23%	71.88%	53.25% to 86.25%

^a^Mean c^td^ values across folds can only be obtained in the original cohort, where the DL survival model was trained.

^b^Evaluation metrics of the best threshold for the DL-based probability of PIRA of the first interval, i.e. ≥3.901%.

95% CI, 95% confidence interval; c^td^: time-dependent concordance index; DL, deep learning; IBS, integrated Brier score; PIRA, progression independent of relapse activity; *P* (PIRA), probability of PIRA (in %); SD, standard deviation.

#### Improvement of conventional survival models with DL-based outputs

The Cox PH model with age only as predictor achieved a Harrell’s C of 0.59. An older age at the first demyelinating attack was significantly associated with a greater risk of PIRA (HR for age, in decades = 1.50 [95% CI 1.07–2.10] *P* = 0.018). The best Cox PH model included age (in decades) at first attack (HR = 1.554 [95% CI 1.096–2.205], *P* = 0.013) and number of brain lesions also at first attack (HR = 1.068 [95% CI 0.992–1.149], *P* = 0.082) (Harrell’s C = 0.62). None of the other baseline variables significantly predicted PIRA in the Cox PH. When we added the min–max normalized DL-estimated cumulative probability of PIRA for the first interval to the best Cox PH model, the Harrell’s C raised up to 0.74 and all three variables remained significant: age (in decades) at first attack: HR = 1.482 (1.041–2.111), *P* = 0.029; number of brain lesion at first attack: HR = 1.093 (1.014–1.179), *P* = 0.021; min–max normalized DL-estimated cumulative probability of PIRA for the first interval: HR = 1.449 (1.306–1.607), *P* < 0.001.

#### Model interpretability: relevance maps

The population-average maps of SHAP values (i.e. relevance maps) revealed that the most relevant areas for predicting PIRA were the frontal and parietal cortex, lateral ventricles, periventricular, frontal and parietal WM. Cortical areas had a more negative relevance, lateral ventricles a more positive one, while a similar relevance value was observed in periventricular WM (Fig. 4).

### External validation analysis

#### Descriptive statistics (validation cohort)

For the external validation analysis, 32 patients fulfilling the inclusion criteria (21 female, mean age at first symptoms: 38.91 [SD 13.46] years) were included, of which 5 (14.29%) had a first PIRA event at a median follow-up time of 1.64 years (range: 1.05; 2.02). The median follow-up time of the external validation cohort was 5.45 (range: 2.65; 6.00) years. At the time of the first attack, those patients who later developed PIRA were slightly older (39.8 versus 38.8 years, *P* = 0.122) and had lower lesion volumes (1.8 versus 8.1 mL, *P* = 0.100) than those not developing it. However, none of these differences reached statistical significance. Median PDDS scores at baseline (0 in both groups, *P* = 0.888), GM fractions (0.48 in both groups, *P* = 0.592) and WM fractions (0.40 and 0.39, *P* = 0.474) were very similar for both groups. At the last visit, patients who had developed PIRA at any time during the follow-up showed higher PDDS scores than patients without PIRA (1 versus 0, *P* = 0.059). Table 3 summarises all the baseline and follow-up data.

**Table 3:** Validation cohort: baseline and follow-up characteristics

	Full cohort	PIRA	Non-PIRA	*P*-value
*N*, (%)	32	4	28	
Baseline characteristics				
Female, *n* (%)	21 (65.62)	4 (100.00)	17 (60.71)	0.122
Age at first attack, years, mean (SD)	38.91 (13.46)	39.75 (2.87)	38.79 (2.70)	0.900
Age (at baseline), years, mean (SD)	39.67 (13.45)	40.62 (2.75)	39.53 (2.70)	0.882
PDDS, median (range)	0 (0; 6)	0 (0; 1)	0 (0; 6)	0.888
Disease duration, years, mean (SD)	0.77 (0.33)	0.87 (0.31)	0.74 (0.35)	0.495
Brain lesion load, ml, mean (SD)	7.27 (7.07)	1.81 (0.33)	8.05 (1.37)	0.100
Brain GM fraction, mean (SD)	0.48 (0.02)	0.48 (0.01)	0.48 (0.01)	0.592
Brain WM fraction, mean (SD)	0.39 (0.02)	0.40 (0.01)	0.39 (0.01)	0.474
Brain parenchymal fraction, mean (SD)	0.87 (0.02)	0.88 (0.004)	0.86 (0.004)	0.141
On DMT at baseline, *n* (%)	20 (62.50)	3 (75.00)	17 (60.71)	0.581
Follow-up characteristics				
Total FU time, years, median (range)	5.45 (2.65; 6.00)	5.70 (4.39; 5.90)	5.42 (2.65; 6.00)	0.593
Disease duration at first PIRA event, years, median (range)	1.64 (1.05; 2.02)	1.64 (1.05; 2.02)		
PDDS at last FU, median (range)	0 (0; 5)	1 (1; 3)	0 (0; 5)	0.059
Annualized relapse rate over FU, median (range)	0.52 (0; 4.72)	0.60 (0.46; 0.89)	0.51 (0; 4.72)	0.503
On DMT at any time during FU, *n* (%)	30 (93.75)	4 (100)	26 (92.86)	0.581

DMT, disease-modifying treatment; FU, follow-up; GM, grey matter; OB, oligoclonal bands; PIRA, progression independent of relapse activity; SD, standard deviation; GMF, grey matter fraction; WMF, white matter fraction; BPF, brain parenchymal fraction.

#### Prediction of PIRA using the best threshold estimated in the original cohort

**Figure 5:**
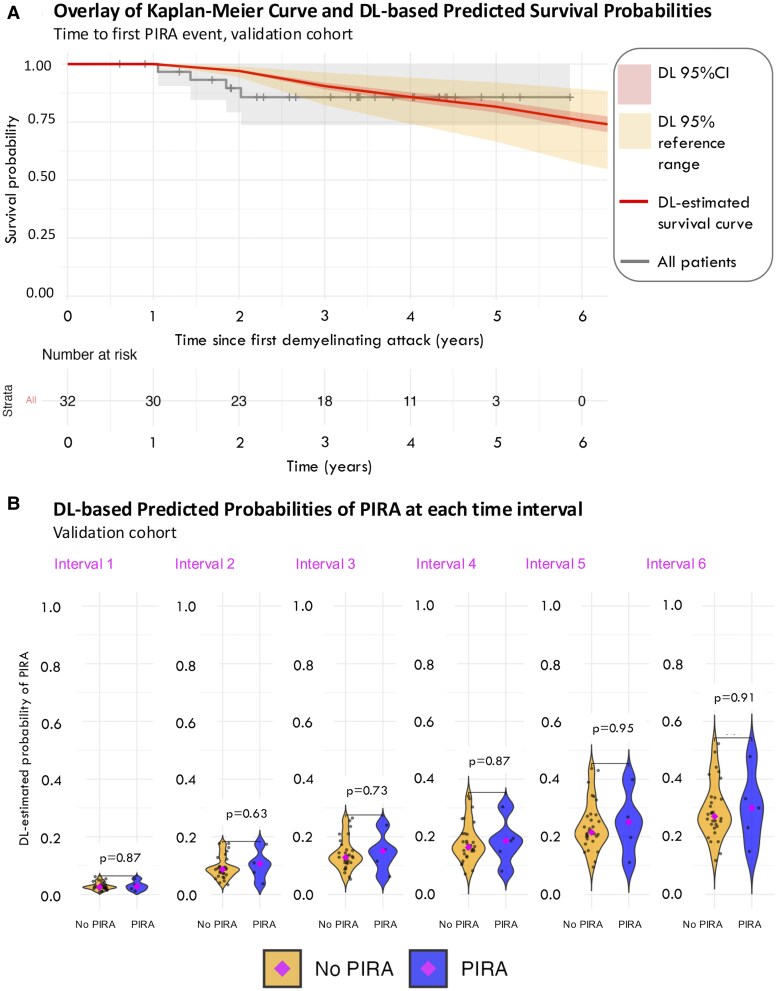
**DL-based predictions in the validation cohort.** (**A**) Overlay of Kaplan–Meier curve (grey) and DL-based survival predictions of PIRA (orange line) in the validation cohort. The darker orange ribbon represents the 95% CI of the estimation, whereas the lighter orange one represents the 95% reference range, i.e. the DL-predicted PIRA survival probabilities in the population, from the 2.5th to the 97.5th percentiles. In the validation cohort, the DL-estimated survival probabilities of PIRA mirrored those of the Kaplan–Meier curve until the 5-year follow-up, when the two curves started to separate. (**B**) DL-based predicted probabilities of PIRA at each time interval. Here, each dot in the graph represents the predicted probability of developing PIRA. As in the original cohort, patients who developed PIRA during the follow-up had, on average, greater DL-predicted risk of developing PIRA at all time points. However, these differences did not reach statistical significance: Mann–Whitney U-test (for comparison of medians): Interval 1: *z* (test statistic, large-sample normal [Z] approximation of the U statistic) = −0.189, *P* = 0.87; Interval 2: z = −0.519, *P* = 0.63; Interval 3: *z* = −0.377, *P* = 0.73; Interval 4: *z* = −0.189, *P* = 0.87; Interval 5: *z* = −0.094, *P* = 0.95; Interval 6: *z* = −0.141, *P* = 0.91. Sample size for all comparisons: *N* = 32. DL, deep learning; PIRA, progression independent of relapse activity.

When the DL model was applied to the external validation cohort, the DL-estimated survival probabilities of PIRA mirrored those of the Kaplan–Meier curve until the 5-year follow-up, when the two curves started to separate (Fig. 5). Furthermore, DL-based predicted probabilities of PIRA at each time interval were, on average, greater in those patients who later developed PIRA during the follow-up than those who never developed it (Fig. 5).

Finally, when the *best VHUH threshold* was applied to the first-interval probability of PIRA in the external validation cohort, we found that it classified patients into future PIRA versus not PIRA with an accuracy of 71.88% (95% CI 53.25% to 86.25%). See Table 2 for full details on evaluation metrics.

## Discussion

In this study, we demonstrate that a DL survival model based solely on conventional brain MRI data acquired at the time of the first demyelinating event can predict the development of PIRA with high accuracy. Our model outperforms previously proposed survival models based on classical statistical methods and may have important implications for clinical practice. This is done through the estimation of interval-specific cumulative probabilities of reaching the event. Of note, of all the estimated interval-specific probabilities of PIRA, we found that the probability for the first time-interval was the most accurate one, significantly improving the performance of a classical Cox proportional hazards model for time to first PIRA, when added as a covariate, independently of age and lesion load at first attack. Furthermore, when the best possible threshold of the first interval-specific probability of PIRA was evaluated, it showed an accuracy of 78% in the original cohort and 71% in the external validation cohort. Although these accuracy values were mainly at the expense of very high specificity values, being sensitivity ones quite low, these results support the generalizability of our DL-based survival model. So, this is the first time, to our knowledge, that a DL-based model is used to predict PIRA, and the first time that PIRA is predicted with such a high accuracy, beyond that obtained, through conventional survival models, when age and lesion burden are accounted for.

The development of the first PIRA event in MS is of paramount importance because it possibly indicates the first clinical evidence of progressive disease in a given patient, even if early in disease course and in the absence of high disability levels.^2-4^ The time of appearance of such first PIRA event has long-term prognostic implications,^4^ and possibly reflects a person’s degree of chronic inflammation and neurodegeneration, both proposed as the main pathological processes underlying PIRA.^18,45,46^ So, an early identification of those people at higher risk of developing PIRA might help design better treatment strategies in clinical practice.

Up to now, only two studies have focused on the prediction of PIRA using clinical, radiological, and demographic characteristics at the time of the first attack or early stages of the disease.^3,4^ Among those features present at the first attack, only an older age has been associated with a greater risk of PIRA.^3,4^ Instead, once the disease is established and patients are far from symptom onset, greater brain lesion volumes^47^ and greater brain^18,47^ and cord^46^ atrophy are also associated with a higher risk of future development of PIRA. The reasons behind the limited ability to predict PIRA at the first attack are unknown. A possible explanation could be that many of the pathological features responsible for PIRA are not yet present at the time of the first attack, and, therefore, their absence does not necessarily indicate that patients will not experience a PIRA event in the coming years. An alternative explanation may be that these features are indeed present at the first attack but remain undetectable with the paraclinical tools we currently have, particularly the routinely acquired MRI. In this context, applying DL to MRI is highly promising, as it can detect features invisible to the naked eye or those not captured by conventional measures, such as lesion count or brain parenchymal fraction.^5^

In our study, we show that the use of DL applied to routinely acquired MRI data may be of great help to identify which patients are at highest risk of developing PIRA. Indeed, as we had hypothesized, our DL-based survival model achieved a high accuracy: its average (across folds—see methods) Harrell’s C score was 0.72, higher than that of survival (Cox) models based only on age or age plus lesion load, all measured at symptom onset. When we looked for the best threshold to be applied to the estimated interval-specific cumulative probabilities of PIRA, we found that PIRA was predicted with a relatively high accuracy of 78% in our cohort—and a bit lower, of 71%, in the external validation cohort. These results are encouraging and suggest that the MRI at the first attack is indeed informative towards the future development of PIRA, beyond classical predictors such as age or lesion load. Furthermore, when the estimated cumulative probability of PIRA for the first time-interval—the one showing the highest accuracy according to ROC analysis—was added as a covariate to the classical Cox model with age and lesion load as covariates (i.e. the *best* Cox model based on our data), the three explanatory variables survived as significant predictors, suggesting independent effects. What is more, the Harrell’s C value increased from 0.62 to 0.74.

It is to be noted, though, that the high accuracy of the DL-based survival model was primarily driven by its high specificity: 94% in the original cohort and 81% in the external validation cohort. This indicates that our model was particularly effective at identifying individuals who would not develop a PIRA event, rather than those who would. This was in line with its high negative predictive value, i.e. 81% in the original cohort and 79% in the external validation one. Instead, the sensitivity was relatively low, i.e. around 25–26% for both cohorts. While our results are encouraging, suggesting that individuals classified as low PIRA risk by the DL survival model might not require drugs targeting chronic inflammation or neurodegeneration from symptom onset, once they become available, future models with higher sensitivity are sorely needed. These models could potentially integrate MRI and laboratory data, such as neurofilament light and, especially, glial fibrillary acidic protein,^45,48-50^ to improve prediction accuracy. Nonetheless, as we had anticipated, our DL model provided better accuracies than any other predictive model built so far to identify patients at highest (and at lowest) risk of developing PIRA. This may have important implications for patient management in routine clinical practice.

Importantly, one of the most innovative aspects of our paper is the survival design of our DL model, implying that we could estimate not only the occurrence of the PIRA event but also when such event occurred. That is, survival models, which are very commonly used in classical statistics (i.e. Cox proportional hazards model), are much less used for DL-based strategies.^34^ Indeed, our study is the first one using that approach for MS data, whereas it has been used in other conditions such as cancer^51^ or coronavirus disease 2019 (COVID-19).^52^

Furthermore, our DL survival model provided relevance maps based on the estimated SHAP values, which inform of the most relevant regions in the input data, i.e. brain regions in our case, for the estimation of the survival probabilities. These relevance maps highlighted the importance of the integrity of the cortical GM for the future development of PIRA, as hypothesized. More specifically, frontal and parietal cortical areas seemed particularly relevant for the prediction of PIRA. This is in line with the findings from Cagol *et al*.,^18^ who found a strong correlation between an increased cortical GM volume loss in people with MS and a concurrent experience of PIRA. Of note, after the frontal and parietal cortical areas, the most relevant regions to predict PIRA were the lateral ventricles, and the periventricular WM, followed by the subcortical WM in the frontoparietal regions. This would support the relevance of WM integrity too for the development of PIRA, as other authors have already pinpointed.^18,47,53^ Periventricular WM (coloured in blue and red) demonstrated relevance in both prognostic directions. This brain region is where T2-lesions most frequently appear, and a higher lesion load has been correlated with disease progression, including reaching PIRA.^47^ The bidirectional nature of these contributions may reflect the influence of patients with and without baseline lesions. In this context, the model might have assigned negative relevance (higher risk of reaching a PIRA) when lesions are present, and positive relevance when they are absent, capturing the distinct impact of lesion presence on the prognosis. However, this needs to be confirmed in future research with larger cohorts. In any case, our research brings to light the potential for DL-based models for not only predicting relevant outcomes in clinical practice but also for investigating the pathogenic mechanisms involved, with potential therapeutic implications.

In our study, we assessed model generalizability through an external validation analysis. So, when we tested the model in a completely unseen, independent cohort, without any re-training or recalibration, we observed that the DL-predicted survival of PIRA was similar to Kaplan–Meier estimates, at least until the 5th year of follow-up, and that the DL-predicted probabilities of PIRA were greater, on average, in those who developed PIRA later on than in those who never developed PIRA. Furthermore, when we applied the *best VHUH threshold* to the external validation cohort, we obtained an acceptable accuracy of 72%, again mainly at the expense of high specificity values, as happened in the original cohort. Although its sensitivity was very low, suggesting future models are required, our findings mean an important step towards the applicability of a DL-based survival model in the clinic.

### Limitations of the study

The main strengths of our study are the unique first-attack patient cohort with an exceptionally long follow-up, the superior accuracy of our DL model predictions compared to classical Cox regression, and the demonstrated generalizability of the model. However, some limitations must also be acknowledged. From a methodological point of view, the sample size and the relatively low density of patients at longer follow-up time intervals made it challenging to estimate survival probabilities as time went by. On the other hand, the estimations for the earliest time intervals seem adequate, according to the high rates of correctly identified PIRA when using the best threshold of the first time-interval cumulative probability of PIRA. An additional limitation is that, to feed our DL model, we only used brain MRI data, whereas the presence of spinal cord atrophy has already proved very relevant for the development of PIRA.^46,54^ Thus, should we have been able to include it in our models, these would have probably reached a better performance, although this deserves further research. Additionally, our first-attack cohort included people with 18–50 years at symptom onset, leaving outside patients with paediatric-onset MS^55^ and late-onset MS.^55-57^ The latter is particularly relevant, given the recent evidence of patients being increasingly older at symptom onset,^56,58^ and the fact that late-onset MS entails a worse prognosis than that MS starting at younger ages.^55-57^ It is possible that this affects model generalizability and surely future DL-based models will need to be explored and validated in older populations. Furthermore, our DL predictive model could not take into account the potential effects of treatment, since its input data was only the MRI at the time of the first attack, before any treatment could be even proposed. In spite of this, its ability to predict PIRA was remarkable. Another important consideration is that when constructing our best Cox model—used as a benchmark and later refined with DL-based estimates—some key clinical factors, such as first attack severity and the functional systems affected at symptom onset, were missing. This could have led to an overestimation of the improvements attributed to DL-based parameters, warranting further investigation. Finally, our validation strategy also had limitations, mainly derived from the small size of the chosen external cohort and the fact that we used self-reported disability scores and self-reported relapses, instead of EDSS and clinician-reported relapses, which might have meant a decrease in our ability to detect PIRA.

## Conclusions

To the best of our knowledge, we present the first DL image-based survival model to predict future prognosis of patients with MS, particularly the development PIRA, from routinely acquired brain MRI scans performed at the time of first demyelinating event. Our model, validated in an independent cohort, predicted who would develop PIRA with acceptable accuracy and improved a classical statistical survival model based only on age and lesion load at symptom onset. Furthermore, our model provided important insights into potential pathogenic mechanisms underlying PIRA development, thanks to the exploitation of explainable AI methods.

Future research directions should include the validation of our current model in larger cohorts with longer follow-ups and its improvement, through accounting for other types of input data collected at symptom onset and the investigation of treatment effects. Moreover, future research should account for the dynamic nature of the disease and find ways of providing dynamic predictions too. Finally, future studies will need to evaluate the actual usefulness in clinical practice, in terms of clinical outcomes and possibly in the context of future drugs able to tackle chronic inflammation, of this and other models that may appear to predict PIRA.

In summary, our findings suggest that the proposed DL survival model holds promise as a tool for managing patients with MS from the onset of symptoms. Future research should explore its potential impact on clinical practice.

## Supplementary Material

fcaf243_Supplementary_Data

## Data Availability

The data used for this study will be available from the senior authors upon reasonable request. All the code generated for the study will be available from GitHub (at https://github.com/cemcat/Predictive_Models_Cemcat).
