# Supplementary material for: Deep learning to predict progression independent of relapse activity at a first demyelinating event
Source: Brain Commun. 2025 Jul 4;7(4):fcaf243. doi: 10.1093/braincomms/fcaf243 (PMC12226453; doi:10.1093/braincomms/fcaf243)
Supplement: fcaf243_Supplementary_Data [file fcaf243_supplementary_data.docx]

**Supplementary Table 1. Recent bibliography (2020-2025) regarding DL-based models in MS**

| **Reference** | | | **N** | **Model** | **Input** | **Output** | **Main performance metrics** |
| --- | --- | --- | --- | --- | --- | --- | --- |
| Tousignant et al.^1^ | 2019 | PMLR. | 465 patients | Deep Learning: CNN | MR (raw) images at study baseline +/- lesion masks as an additional input | Confirmed disease progression at 1-year follow-up (binary outcome) | Accuracy: 70.1% |
| Taloni et al.^2^ | 2022 | Int J Mol Sci. | 181 patients | Deep Learning: CNN | T1-weighted brain lesions | EDSS increase by ≥ 1.5, 1.0, 0.5 at follow-up | AUC: 0.81 |
| Storelli et al.^3^ | 2022 | Invest Radiol. | 373 patients | Deep Learning: CNN | Coregistered T1-weighted and T2-weighted baseline images in MNI space, as well as baseline clinical and demographic data | Clinical worsening on (i) EDSS, (ii) SDMT, (iii) EDSS or SDMT at 2-year follow-up (binary outcome) | Accuracy for: EDSS worsening: 83.3% SDMT worsening: 67.7% EDSS or SDMT worsening: 85.7% |
| Coll et al.^4^ | 2023 | Neuroimage Clin. | 319 patients | Deep Learning: CNN | T1-weighted, T2-weighted brain lesions | EDSS (binary outcome: < or ≥ 3) | Accuracy: 0.79 |
| Zhang et al.^5^ | 2023 | BMC Med Inform Decis Mak. | 300 patients | Deep Learning: Modified encoder-decoder | Clinical notes, patient demographics, medical diagnoses, prescribed medications, and administered treatments, structured EHR data (laboratory test measurements, vital sign observations, medication administrations, demographic information, and MRI data: pre-contrast and post-contrast T1-weighted sequences, T2-weighted sequences, proton density-weighted sequences, and FLAIR sequences | EDSS > 4; 6; 7 | ROC AUC = 0.84 (when using all input data available) |
| Mayfield et al.^6^ | 2024 | J Imaging Inform Med. | 703 patients | Deep Learning: ViViT, ViT-LSTM | T2-weighted, T1- weighted, and T1- weighted plus Contrast spinal cord MRI (no lesion segmentation) | EDSS (trinary outcome: Mild, moderate, severe) | The VGG16-LSTM predicted trinary classification of EDSS score in 6 years with 0.74 AUC versus the ViViT with 0.84 AUC. While the less time-dependant VGG16-LSTM outperformed ViViT when patients with only 2 years of MRIs (n = 94) (0.75 AUC versus 0.72 AUC, respectively). |
| Montolío et al.^7^ | 2024 | Acta Ophthalmol. | 72 patients | Deep Learning: Feedforward neural network | mGCL thickness features from OCT | Disability progression (binary outcome) | Accuracy: 0.82 |

**Supplementary Table 1 (footnote).** *Abbreviations*: AUC: area under the curve; CNN: convolutional neural network; EDSS: expanded disability status scale; HER: electronic health records; FLAIR: fluid attenuated inversion recovery; mGCL: macular ganglion cell layer; OCT: optical coherence tomography; ROC (curve): receiver operating characteristic (curve); SDMT: symbol digit modalities test.

**Supplementary Table 2. Glossary related to DL-based and survival analyses**

| **Term (in alphabetical order)** | **Meaning** |
| --- | --- |
| **Classical survival models** | |
| aHR | Hazard ratio (HR) is a measure of the relative risk of an event (e.g., PIRA, in our case) occurring in one group compared to another (for categorical predictors), or per each unit increase in the predictor variable (for continuous predictors), over time. It is commonly used in survival analysis. Adjusted hazard ratio (aHR) has the same meaning as the HR, but when the model is adjusted (generally for confounders). |
| Cox PH | The Cox Proportional Hazards (Cox PH) model is a semi-parametric survival analysis model (based on classical statistics) used to estimate the effect of covariates on the hazard (risk) of an event occurring over time. While it has been widely used in epidemiological research, due to its simplicity, it has important considerations/limitations that need to be taken into account: the proportional hazards assumption must hold, i.e., the relative effect of a given covariate on the risk of an event occurring must be constant over time; the baseline hazard is not estimated by the model (i.e., it remains non-parameterised). |
| Harrell's concordance index or c-index | The Harrell’s C index is a measure of how well a survival model can discriminate between those having and those not having the event. More specifically, it quantifies how well the predicted risks match the actual event times. Harrell’s C values close to 1 suggest a very good (perfect) match or prediction, while values equal to 0.5 indicate random guessing. |
| Kaplan-Meier curves | A Kaplan-Meier (KM) curve is a stepwise survival plot that estimates the probability of an event-free survival over time. It visualizes time-to-event data, accounting for censoring, and compares survival rates between groups in medical and survival analysis. |
| Survivor function | The survivor function (or survival function) represents the probability that an individual survives beyond a given time 𝑡. In our case, it would represent the probability that a patient with MS survives (i.e., does not develop PIRA) beyond a given time 𝑡. |
| **Deep learning models** | |
| CNN | Deep learning architecture designed for processing grid-like data (e.g., images). It uses convolutional layers to extract spatial features, enabling efficient pattern recognition and classification. This makes CNNs very powerful tools to predict any types of outcomes, including clinical ones. |
| CNN’s depth | The depth of a CNN refers to the total number of *learnable layers* in the network, including convolutional layers, fully connected layers, and sometimes batch normalization layers. It does not count pooling or activation layers. |
| CNN’s resolution | The resolution of a CNN refers to the spatial dimensions (height × width) of the input feature maps after having passed through the convolutional and pooling layers of the network. Higher resolution CNNs preserve details but increase computational cost and require more data to train the network. |
| CNN’s width | The width of a CNN refers to the number of filters (or neurons) in each convolutional layer. Wider networks (more filters per layer) capture more complex patterns in images and improve feature extraction and representation capacity. However, they are more computationally expensive and require more data to train the network. |
| DL | Deep learning is a subset of machine learning using multi-layered artificial neural networks to automatically learn patterns from data, enabling tasks like image recognition, natural language processing, and medical diagnosis. |
| EfficientNet-b0 | EfficientNet-b0 is the baseline model of the EfficientNet family, a deep convolutional neural network optimised for accuracy and efficiency. |
| Fold | A fold refers to a subset of data used in cross-validation (i.e., k-fold cross-validation analysis). This type of analysis is used to evaluate a model's performance by splitting the dataset into k equal parts (folds). Each fold acts as a validation set once, while the remaining k−1 folds are used for training. This process repeats k times, ensuring the model is tested on all data points. |
| Fully connected layer | A fully connected layer is a layer in a neural network where each neuron is connected to every neuron in the previous and next layers. It is commonly used in the final stages of CNNs. |
| GAP | Global Average Pooling (GAP) is a downsampling operation used in CNNs which consists of averaging each feature map across its spatial dimensions. So, the introduction of a GAP layer helps reduce the resolution of the network. |
| IBS | The integrated Brier score (IBS) quantifies the mean square difference between the predicted survival probabilities and the observed event-time. The lower it is, the better the performance of the model will be. |
| ImageNet | ImageNet is a large-scale image dataset used for computer vision research, containing over 14 million labeled images across 1,000 object categories. It is widely used for training and benchmarking deep learning models, especially in image classification tasks. |
| MBConv | A Mobile Inverted Bottleneck Convolution (MBConv) is an efficient convolutional block designed to improve computational efficiency in deep learning models. |
| SHAP maps | SHAP maps refer to visual representations derived from SHAP (SHapley Additive exPlanations) values, which are used mainly in deep learning to interpret model predictions. More specifically, SHAP maps highlight which parts of an image influence the model’s decision the most, providing insight into why a neural network made a particular classification or regression prediction. |
| Sigmoid function | The sigmoid function is an activation function commonly used in deep learning, particularly in binary classification tasks. It maps any real-valued input into a range between 0 and 1, making it useful for probability-based predictions. |
| Training | Training in deep learning refers to the iterative process of optimising a neural network by adjusting its parameters (weights and biases) so that it learns to map inputs to outputs effectively, i.e., it learns to predict the outcome based on the input data.  This process involves feeding data (i.e., input data) through the model, comparing predictions to ground truth labels, and updating parameters using optimisation techniques. |

**Supplementary Table 2 (footnote).** *Abbreviations*: aHR: adjusted hazard ratio; AUC: area under the ROC curve; CNN: convolutional neural network; Cox PH: Cox proportional hazards (model); DL: deep learning; GAP: Global Average Pooling; IBS: Integrated Brier score; MBConv: Mobile Inverted Bottleneck Convolution; MS: multiple sclerosis; PIRA: progression independent of relapse activity; ROC: Receiver operating characteristic; SHAP: SHapley Additive exPlanations;

**Supplementary Figure 1. Distribution of DL-based interval-specific cumulative probabilities of reaching a first PIRA event**


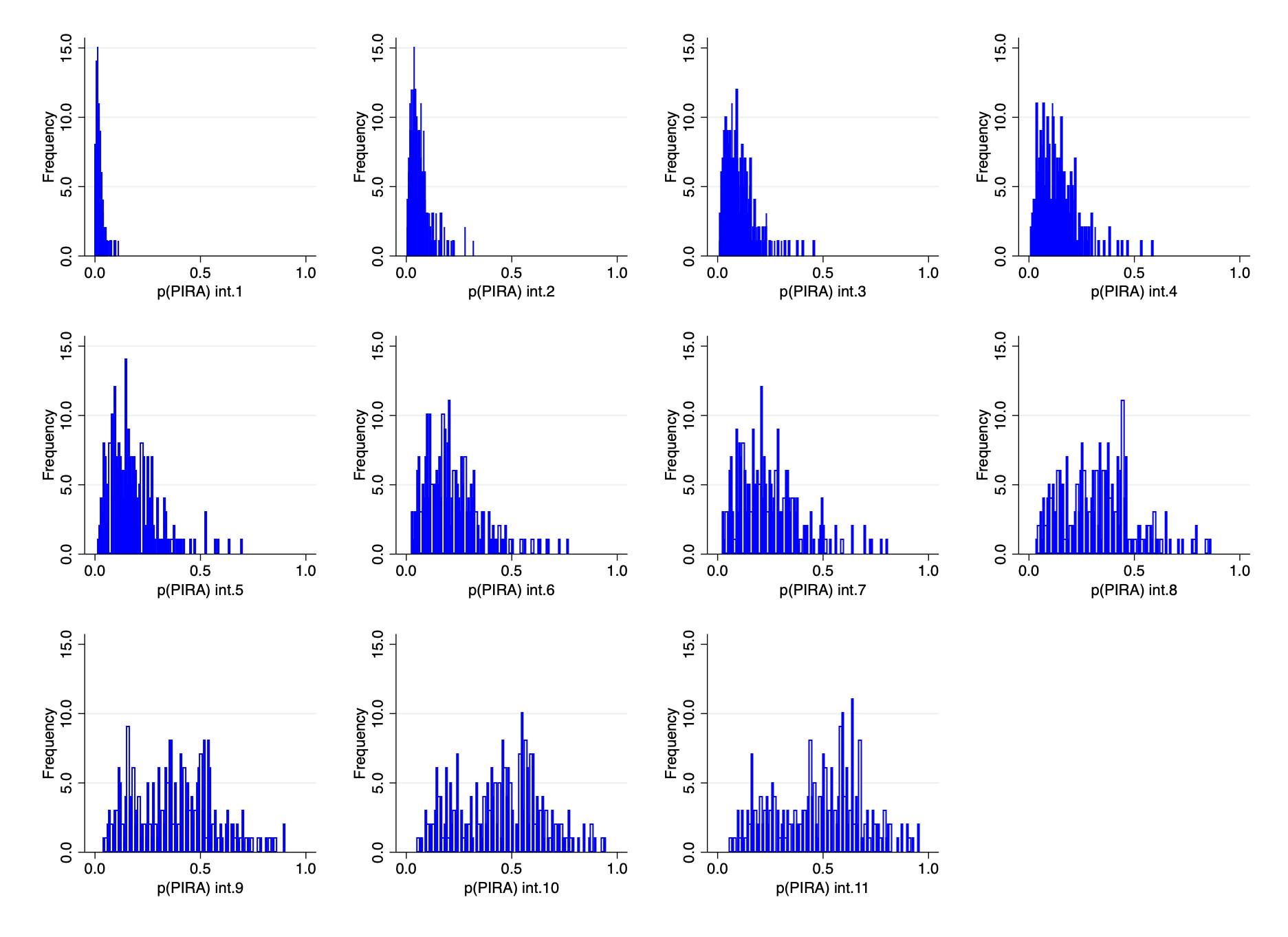


**Supplementary Figure 1.** Distribution of DL-based interval-specific cumulative probabilities of reaching a first PIRA event. Each data point represents an individual patient’s predicted probability of reaching the outcome, i.e., a first PIRA event after the first demyelinating attack, at the different study intervals, according to the deep learning survival model. The number of data points in each histogram is exactly the same (N = 259), corresponding to the size of the original cohort. *Abbreviations*: DL: deep learning; int.: interval; PIRA: progression independent of relapse activity.

**Supplementary reference list**

1. Tousignant A, Lemaître P, Precup D, Arnold DL, Arbel T. Prediction of Disease Progression in Multiple Sclerosis Patients using Deep Learning Analysis of MRI Data. *Proceedings of Machine Learning Research*. 2019;(102):483-492.

2. Taloni A, Farrelly FA, Pontillo G, et al. Evaluation of disability progression in multiple sclerosis via magnetic-resonance-based deep learning techniques. *Int J Mol Sci*. 2022;23(18):10651. doi:10.3390/ijms231810651

3. Storelli L, Azzimonti M, Gueye M, et al. A deep learning approach to predicting disease progression in multiple sclerosis using magnetic resonance imaging. *Invest Radiol*. 2022;57(7):423-432. doi:10.1097/rli.0000000000000854

4. Coll L, Pareto D, Carbonell-Mirabent P, et al. Deciphering multiple sclerosis disability with deep learning attention maps on clinical MRI. *NeuroImage Clin*. 2023;38(103376):103376. doi:10.1016/j.nicl.2023.103376

5. Zhang K, Lincoln JA, Jiang X, Bernstam EV, Shams S. Predicting multiple sclerosis severity with multimodal deep neural networks. *BMC Med Inform Decis Mak*. 2023;23(1):255. doi:10.1186/s12911-023-02354-6

6. Mayfield JD, Murtagh R, Ciotti J, Robertson D, Naqa IE. Time-dependent deep learning prediction of multiple sclerosis disability. *J Imaging Inform Med*. 2024;37(6):3231-3249. doi:10.1007/s10278-024-01031-y

7. Montolío A, Cegoñino J, Garcia-Martin E, Pérez del Palomar A. The macular retinal ganglion cell layer as a biomarker for diagnosis and prognosis in multiple sclerosis: A deep learning approach. *Acta Ophthalmol*. 2024;102(3). doi:10.1111/aos.15722
